# Effect of Single High-Dose Vitamin D_3_ Supplementation on Post-Ultra Mountain Running Heart Damage and Iron Metabolism Changes: A Double-Blind Randomized Controlled Trial

**DOI:** 10.3390/nu16152479

**Published:** 2024-07-31

**Authors:** Błażej Stankiewicz, Jan Mieszkowski, Andrzej Kochanowicz, Paulina Brzezińska, Bartłomiej Niespodziński, Tomasz Kowalik, Tomasz Waldziński, Konrad Kowalski, Andżelika Borkowska, Joanna Reczkowicz, Ludmiła Daniłowicz-Szymanowicz, Jędrzej Antosiewicz

**Affiliations:** 1 Department of Theory and Methodology of Physical Education and Sport, Faculty of Health Sciences and Physical Education, Kazimierz Wielki University, 85-064 Bydgoszcz, Poland; blasta@ukw.edu.pl (B.S.); tom.kow@ukw.edu.pl (T.K.); 2 Department of Gymnastics and Dance, Gdańsk University of Physical Education and Sport, 80-336 Gdańsk, Poland; andrzej.kochanowicz@awf.gda.pl (A.K.); paulina.brzezinska@awf.gda.pl (P.B.); 3 Faculty of Physical Education and Sport, Charles University, 16-252 Prague, Czech Republic; 4 Department of Biological Foundations of Physical Education, Faculty of Health Sciences and Physical Education, Kazimierz Wielki University, 85-064 Bydgoszcz, Poland; bartlomiej.niespodzinski@ukw.edu.pl; 5 Faculty of Health Sciences, University of Lomza, 18-400 Łomża, Poland; twaldzinski@al.edu.pl; 6 Department of Bioenergetics and Physiology of Exercise, Medical University of Gdańsk, 80-211 Gdańsk, Poland; konrad.kowalski@masdiag.pl (K.K.); andzelika.borkowska@gumed.edu.pl (A.B.); joanna.reczkowicz@gumed.edu.pl (J.R.); 7 Department of Cardiology and Electrotherapy, Medical University of Gdansk, 80-214 Gdansk, Poland; ludmila.danilowicz-szymanowicz@gumed.edu.pl

**Keywords:** iron, ferritin, ultramarathon, erythroferrone, erythropoietin, vitamin D

## Abstract

Exercise-induced inflammation can influence iron metabolism. Conversely, the effects of vitamin D_3_, which possesses anti-inflammatory properties, on ultramarathon-induced heart damage and changes in iron metabolism have not been investigated. Thirty-five healthy long-distance semi-amateur runners were divided into two groups: one group received 150,000 IU of vitamin D_3_ 24 h prior to a race (*n* = 16), while the other group received a placebo (*n* = 19). Serum iron, hepcidin (HPC), ferritin (FER), erythroferrone (ERFE), erythropoietin (EPO), neopterin (NPT), and cardiac troponin T (cTnT) levels were assessed. A considerable effect of ultramarathon running on all examined biochemical markers was observed, with a significant rise in serum levels of ERFE, EPO, HPC, NPT, and cTnT detected immediately post-race, irrespective of the group factor. Vitamin D_3_ supplementation showed a notable interaction with the UM, specifically in EPO and cTnT, with no other additional changes in the other analysed markers. In addition to the correlation between baseline FER and post-run ERFE, HPC was modified by vitamin D. The ultramarathon significantly influenced the EPO/ERFE/HPC axis; however, a single substantial dose of vitamin D_3_ had an effect only on EPO, which was associated with the lower heart damage marker cTnT after the run.

## 1. Introduction

Prolonged exercise is associated with an increased inflammation process that may limit physical performance and directly affect the health of sportsmen [1]. Intensive proinflammatory cytokine secretion affects working muscle metabolism and influences the functioning of many other tissues and organs [2]. Moreover, data show that the systematic post-exercise response can be modulated by iron status [3]. Iron homeostasis is an essential factor for maintaining human health. Both iron deficiency and excess may contribute to the development of many diseases [4]. Conversely, both recreational and sports activity lead to a decline in body iron stores, which is considered positive if the changes are in the physiological range [5]. One of the major regulatory proteins in iron metabolism is hepcidin (HPC). Exercise has been shown to upregulate IL-6, which stimulates HPC biosynthesis. Elevated blood HPC impedes the absorption of iron from the gastrointestinal tract and diminishes its release from the liver and other tissues into the bloodstream, leading to a reduction in serum iron levels. As iron can augment the inflammation process, decreasing its concentration as a result of IL-6/HPC action may be beneficial. Serum HPC can also be influenced by some other factors, like changes in iron concentration and hormones like erythropoietin (EPO) and erythroferrone (ERFE). Ischemia, which can occur during exercise, increases EPO, which, in turn, stimulates ERFE. ERFE is secreted by erythroblasts after stimulation with EPO via the JAK2-STAT5 pathway, leading to the inhibition of HPC synthesis by hepatocytes. This ultimately results in an increase in iron absorption [6]. Thus, the EPO/ERFE axis gives opposite signalling to inflammatory cytokines. Moreover, changes in the iron-dependent inflammatory response in some situations can be induced by myocardium muscle damage, especially during prolonged exercise and post-exercise-induced inflammation [7,8]. From among many non-specific markers associated with immune activation during inflammation whose concentration increases when the cellular immune defence is stimulated in a wide range of conditions, neopterin (NPT) may deserve special attention. Its secretion is generated by interferon-γ-activated macrophages and has been widely tested for the diagnosis, prognosis, and assessment of many inflammation-associated conditions. Due to this fact, NPT has been routinely measured and has shown promise as an indicator of over-training syndrome in numerous sport-associated conditions, e.g., during high-intensity and prolonged distance cycling, team sport competition, and others [9,10].

Exercise has been demonstrated to influence vitamin D metabolism, primarily evidenced by an elevated 25(OH)D_3_ serum level. Considering that many athletes are vitamin D-deficient, exercise-induced increases in 25(OH)D_3_ may be limited in such persons. Studies both on athletes and patients proved that vitamin D has anti-inflammatory effects [11]; thus, its influence on iron metabolism can be expected. Conversely, EPO, a hormone involved in iron metabolism, had been shown to have a cardioprotective effect [12]. Numerous studies indicate that prolonged exercise can cause heart damage, as evidenced by increased levels of serum cardiac troponin T (cTnT) [8]. This is a significant issue because even slight increases in troponin I levels after long-distance walking have been shown to independently predict higher mortality and cardiovascular events [13]. Nevertheless, information on the interplay between iron metabolism, vitamin D, and exercise-induced heart damage is quite scarce. A significant dose of vitamin D has been shown to down-regulate resting plasma hepcidin without influencing proinflammatory cytokines [14]. Therefore, it remains uncertain whether the effects of vitamin D on iron metabolism are mediated by reductions in inflammation or by directly influencing HPC expression [14]. Moreover, it has been proven that there is an inverse correlation between serum 25(OH)D_3_ concentration and serum neopterin level [15], suggesting that low vitamin D concentration may be linked to heightened inflammatory processes and greater heart damage during exercise.

Thus, the purpose of this research was to examine the effects of a significant amount of vitamin D_3_ supplementation on serum markers of iron metabolism, such as HPC, ferritin (FER), ERFE, EPO, and NPT, in healthy ultra mountain runners. Moreover, we hypothesised that vitamin D treatment would mitigate ultramarathon-induced heart damage, as evidenced by elevated cTnT concentrations.

## 2. Materials and Methods

### 2.1. Experimental Overview

The presented research is an extension of scientific work focused on the effects of a single high dose of vitamin D_3_ on vitamin D metabolites in ultramarathon runners. It is a part of the project “Vitamin D as a Factor Modifying Adaptation to Exercise”. However, it is important to briefly recall its main objectives at this point.

The conducted research was a double-blind, randomised controlled trial with parallel groups, which included a supplementation and a placebo (control) group. The supplementation involved administering a single substantial dose of vitamin D_3_.

On the initial visit (performed on the eighteenth and nineteenth of July 2018), information on participants’ age, body composition, and height was gathered. Additionally, a professional physician performed medical examinations and the participants consented or did not consent to participate in the study. Venous blood samples for the specific biochemic analyses were collected 24 h before, immediately after, and 24 h after the ultramarathon run. Vitamin D status and serum iron metabolism markers were assessed at the Sport University in Gdańsk (Gdańsk, Poland).

### 2.2. Study Population

This research included 35 semi-professional male ultramarathon runners who took part in the 2018 Lower Silesian Mountain Runs Festival Ultra Marathon Race. Prior to the event, the participants filled out an online survey. They were then randomly assigned to either the experimental group (supplemented, S; *n* = 16) or the placebo group (control, C; *n* = 19). The characteristics of the participants can be found in Table 1, and they were part of a broader research project, as discussed in previous work by Mieszkowski et al. [16].

Participants eligible for inclusion were male runners aged 30 years or older with a history of at least five ultramarathon finishes over distances exceeding 42 km. They had to be healthy, non-smokers, with no additional drug intake, and without any current vitamin D or antioxidant supplementation.

The exclusion criteria included individuals with physical or mental impairments, those currently undergoing treatment for psychiatric disorders, or those with alcohol or substance abuse issues who were unable or unwilling to comply with the study evaluations. Participants were also excluded if they suffered from severe inflammation diseases or inflammation conditions.

Additionally, the included runners completed a survey to outline the methods and training loads during their training period (general preparation and pre-race preparation phases) (Table 2). The general preparation period spanned 12 weeks leading up to the pre-start phase before the Lądek Zdrój Mountain Run. According to the survey, the pre-start period lasted between 8 and 12 weeks. All ultramarathon runners reported using pulse monitoring devices to control their training process (HR measurement). Additionally, to determine the approximate level of VO_2_ max as a measure of physical readiness for the competition, participants were asked to perform a 12 min Cooper test at maximum intensity approximately 2 weeks before the race. The results from this test were used to calculate the VO_2_ max indicator (using an indirect method) using the formula VO_2_ max = (22.351 × distance covered in kilometres) − 11.288 (Table 3). Participants abstained from consuming stimulants, including alcohol, caffeine, chocolate, guarana, tea, or theine, one week before the experiment and throughout all testing periods.

Furthermore, the included runners were given equal meal plans according to a standardised diet that was customised for their age group and level of physical activity. They were instructed to follow these diets on the measurement days. 

Before the start of the study, the research protocol was approved (decision no. KB-24/16) by the Bioethics Committee for Clinical Research at the Regional Medical Chamber in Gdańsk. All study steps were performed in accordance with the Declaration of Helsinki. Participants provided informed written consent before enrolling in the study. They were briefed on the study procedures but were not informed of the study’s rationale and aim, ensuring they were unaware of the potential effects of supplementation. The study was registered as a clinical trial (NCT03417700).

Sample size power analysis was conducted using GPower ver. 3.1.9.2. software (Franz Faul, Universität Kiel, Kiel, Germany), and calculated as 28 participants.

### 2.3. Prolonged Run—Mountain Ultramarathon

All runners took part and finished the Lower Silesian Mountain Run Festival 2018 (Lądek Zdrój; Lower Silesian Voivodeship, Poland). The race started at 6:00 p.m and included a maximum course length of 240 km, with the highest altitude reaching approximately 1425 m above sea level and the lowest altitude at around 261 m above sea level. The total altitude range covered was approximately 1164 m, with a total ascent and descent of 7670 m, and temperatures varying from 18 °C to 4 °C.

### 2.4. Vitamin D_3_ Administration

Runners in the S group received a single 150,000 IU dose of vitamin D_3_ in a 10 mL vegetable oil solution 24 h before the ultramarathon. The C group was given a placebo solution of the same volume. The placebo solution was designed to have the same taste (anise), consistency, and colour as the vitamin D_3_ oil solution. Neither the participants nor the researchers knew the group assignments, as the supplementation and placebo solutions were provided in sealed sintered glass bottles labelled with randomly assigned numbers.

### 2.5. Sample Collection Protocol

Blood samples were collected at specified timepoints: 24 h before the run, instantly after the run (within 5 min of finishing), and 24 h post-run. Blood was drawn into serum S-Monovette tubes (S-Monovette^®^ Sarstedt AG&Co, Nümbrecht, Germany). Furthermore, the serum was extracted, divided into 500 µL aliquots, and stored at −80 °C until analysis, for a maximum duration of 6 months.

Liquid chromatography–tandem mass spectrometry (Shimadzu Nexera X2 UHPLC, Shimadzu, Japan) paired with an 8050 triple quadrupole detector (Shimadzu) was used for the quantitative analysis of vitamin D. Serum proteins were first precipitated and derivatised. LabSolutions LCGC was used to collect, process, and quantify the raw data. The concentrations of the vitamin D metabolites 25(OH)D_3_ and 24,25(OH)_2_D_3_ were assessed at all sampling timepoints.

The levels of the following serum iron metabolism markers were determined: free serum iron, HPC, FER, ERFE, EPO, and NPT.

Total iron, HPC, FER, TIBC, and UIBC were analysed at Anmed Medical Laboratory in Lądek Zdrój, Poland. The serum EPO tests were conducted using a MAGPIX fluorescence detection system (Luminex Corp., Austin, TX, USA) with dedicated assays. Serum ERFE and NPT levels were determined with the ELISA method following the manufacturer’s instructions using a Human Erythropoietin Quantikine ELISA Kit (R&D Systems, Inc., USA) and Human Neopterin ELISA Kit (FineTest Biotech Inc., Wuhan, Hubei, China).

Potential changes in the haematocrit values were accounted for in the data presentation.

### 2.6. Statistical Analysis

All analysed variables were reported as the means ± standard deviation (SD). A one-way ANOVA was conducted to evaluate variations in attributes among groups. Additionally, a two-way ANOVA with repeated measures (2 × 3) was used to examine the impact of the ultramarathon at three timepoints (24 h before, immediately after, and 24 h post-run) on the 25(OH)D_3_ and 24,25(OH)_2_D_3_ levels, HPC, EPO, NPT, and ERFE levels, and iron metabolism markers, in relation to vitamin D_3_ supplementation (group: S vs. C). Similarly, a two-way ANOVA with repeated measures (2 × 2) was employed to assess the impact of the ultramarathon (baseline, 24 h after the run) on the cTnT levels in both groups. In cases of identified significant interactions, Tukey’s post hoc test was conducted. The Shapiro–Wilk and Levene tests were used to verify the assumption of normality and homogeneity of variances.

To examine the interaction between changes in serum HPC, EPO, NPT, and ERFE levels and the baseline iron metabolism markers induced by the ultramarathon, Pearson’s (r) correlation was used. Eta-squared statistics (η^2^) were employed to assess the effect size, with thresholds of 0.01, 0.06, and 0.14 representing small, moderate, and large effects, respectively. All calculations and graphics were generated in Statistica 12 (StatSoft, Tulsa, OK, USA). Statistical significance was set at *p* ≤ 0.05.

## 3. Results

Significant increases in serum levels of vitamin D_3_ metabolites were observed immediately after and 24 h post-ultramarathon in both study groups. However, when considering the haematocrit-adjusted data, the changes observed in the S group immediately after the run (25(OH)D_3_, Δ = 46.67 ± 14.87 ng/mL; 24,25(OH)_2_D_3_, Δ = 3.34 ± 1.48 ng/mL) were significantly greater than those in the control group (25(OH)D_3_, Δ = 33.06 ± 8.08 ng/mL, *p* ≤ 0.01; 24,25(OH)_2_D_3_, Δ = 2.51 ± 0.94 ng/mL, *p* ≤ 0.05).

The serum levels of 25(OH)D_3_ and 24,25(OH)_2_D_3_ at baseline were comparable between the two groups.

**Figure 1:**
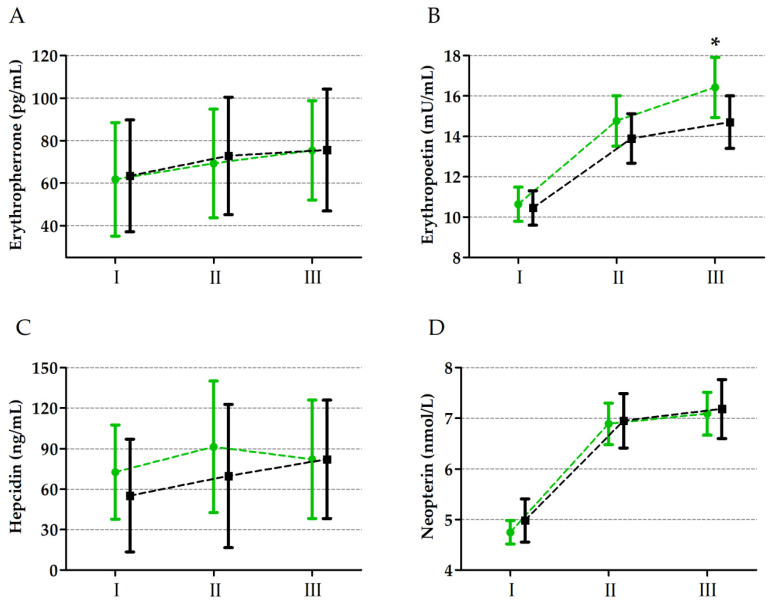
Changes in erythroferrone (**A**), erythropoietin (**B**), hepcidin (**C**), and neopterin (**D**) levels induced by ultramarathon in runners supplemented with vitamin D_3_ (green colour) and runners supplemented with placebo (black colour). Sampling: I, 24 h before the run; II, immediately after the run; III, 24 h after the run. * Significant difference vs. placebo (control) group 24h after the run detected at *p* ≤ 0.01.

A significant effect of the ultramarathon on all analysed biochemical markers was observed, as indicated by the two-way ANOVA results for ERFE, EPO, HPC, and NPT levels, which are presented in Table 4 and Figure 1. Regardless of the group factor, there was a significant increase in serum concentrations of ERFE (13.6%, *p* ≤ 0.01), EPO (35.2%, *p* ≤ 0.01), HPC (26.2%, *p* ≤ 0.01), and NPT (43.7%, *p* ≤ 0.01) immediately after the run compared to the baseline values. Furthermore, ERFE (7.1%, *p* ≤ 0.05), EPO (5.8%, *p* ≤ 0.05), and NPT (3.2%, *p* ≤ 0.05) levels continued to rise 24 h post-run compared to the levels recorded immediately after the run.

EPO showed a significant interaction between the group factor and the ultramarathon. The post hoc analysis revealed that EPO concentrations were significantly higher in the vitamin D_3_-supplemented group compared to the placebo group 24 h after the run (7.9%, *p* ≤ 0.05).

Pearson’s correlation results indicated a significant positive relationship in the placebo group between changes in ERFE concentration immediately after (*p* ≤ 0.05) and 24 h post-run (*p* ≤ 0.05) and baseline ferritin levels (Table 5). Additionally, baseline FER levels in the placebo group showed a significant positive correlation with changes in HPC levels immediately after the run (*p* ≤ 0.05).

Conversely, in the supplemented group, baseline FER levels exhibited an inverse correlation with changes in HPC levels immediately after the run in the supplemented group (*p* ≤ 0.05). Furthermore, baseline iron levels in the placebo group demonstrated a significant relationship with changes in NPT concentration 24 h after the run (*p* < 0.05).

**Figure 2:**
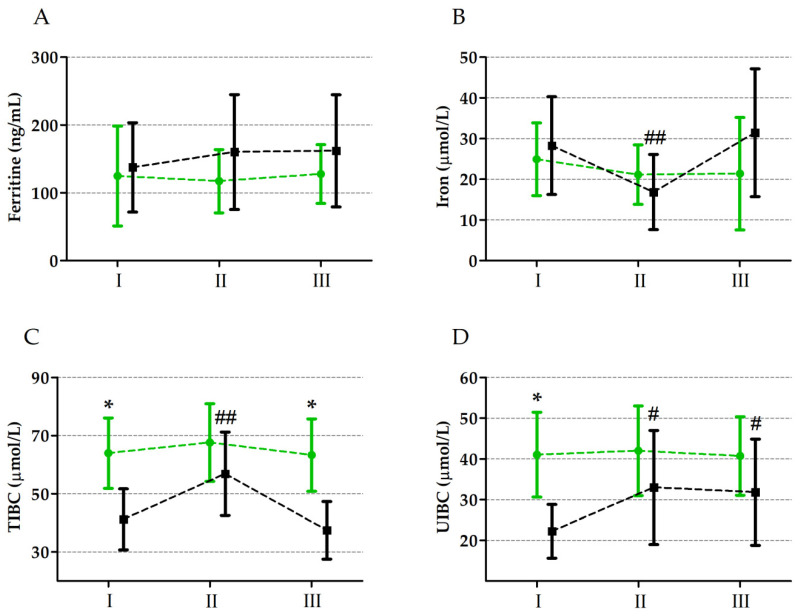
Changes in ferritin (**A**), iron (**B**), TIBC—total iron-binding capacity (**C**), and UIBC—unsaturated iron-binding capacity (**D**) levels induced by ultramarathon in runners supplemented with vitamin D_3_ (green colour) and with placebo (black colour). Sampling: I, 24 h before the run; II, immediately after the run; III, 24 h after the run. * significant difference between runners supplemented with vitamin D_3_ and placebo (control) group at a particular timepoints at *p* ≤ 0.01; # significant difference from baseline value in a particular group at *p* ≤ 0.01; ## significant difference from baseline value and 24 h after the run in a particular group at *p* ≤ 0.01.

The two-way ANOVA results for changes in the ferritin, iron, total iron-binding capacity (TIBC), and unsaturated iron-binding capacity (UIBC) levels induced by the ultramarathon are presented in Table 6 and Figure 2. The ultramarathon had a significant effect on iron, TIBC, and UIBC levels. TIBC and UIBC levels significantly increased after the run by 18.3% (*p* ≤ 0.01) and 15.6% (*p* ≤ 0.05), respectively, while iron levels significantly decreased by 29.2% (*p* ≤ 0.01) compared to the baseline values. The analysis of variance also showed a significant influence of the group factor on TIBC and UIBC levels, which were higher in the supplemented group regardless of the ultramarathon. The post hoc analysis revealed a significant interaction between the ultramarathon and group factors, with the placebo group showing a significant increase in TIBC (37.8%, *p* ≤ 0.01) and UIBC (42.0%, *p* ≤ 0.01) and a significant decrease in iron levels (38.5%, *p* ≤ 0.01) immediately after the ultramarathon.

The two-way ANOVA of changes in cTnT levels induced by the ultramarathon run is presented in Table 7.

## 4. Discussion

Previous research demonstrated that a single oral dose of vitamin D_3_ (150,000 IU) significantly reduced inflammation induced by ultramarathon running [16]. The changes in IL-6, resistin, and IL-10 were attenuated in vitamin D_3_-supplemented athletes. In the present study, based on a number of studies demonstrating that inflammation influences iron metabolism, we hypothesise that vitamin D_3_ can mediate the changes in iron metabolism. The primary aim of this study was to investigate the impact of ultramarathon running on hormones involved in iron metabolism, such as HPC, EPO, and ERFE, and to determine whether vitamin D_3_ supplementation would modify this process. While HPC levels increased after the run, vitamin D_3_ had no effect on this change. This is an unexpected observation, as previously, we observed that vitamin D_3_ significantly blunted ultramarathon-induced increases in IL-6 [16]. Unexpectedly, here, we observed that vitamin D supplementation did not influence the exercise-induced increase in serum NPT. Additionally, IL-6 has been shown to stimulate HPC synthesis [17]; thus, it could be expected that in vitamin D_3-_supplemented athletes, the change will be less pronounced. Moreover, studies on cell-cultured hepatocytes demonstrated that 25-hydroxyvitamin D or active 1,25-dihydroxyvitamin D reduced HPC mRNA expression by half [18]. In a related pilot study with healthy volunteers, the same authors observed a significant decrease in circulating HPC levels within 24 h after supplementation with a single oral dose of vitamin D (100,000 IU vitamin D_2_) [18]. These findings suggest that vitamin D can affect blood HPC levels; however, this effect was not observed in our study. Unfortunately, we cannot compare the effects of vitamin D_3_ supplementation in resting conditions as vitamin D_3_ was given just after the first blood sampling, which was 24 h before the run. HPC synthesis has been demonstrated to be inhibited by ERFE, a hormone synthesised by nucleated red blood cells [19]. Its serum concentration increases after the run; thus, downregulation of HPC could be expected, but an increase was observed instead. Again, no effects of vitamin D_3_ on ERFE were observed. Conversely, the synthesis of ERFE is stimulated by EPO [19] and a consistent increase in ERFE was associated with an increase in EPO after the run. In our study, the changes in EPO were higher in the S group 24 h after the run. Serum iron is another factor which can influence serum HPC concentration. A decrease in serum iron can be a result of HPC action, which is known to block ferroportin, a protein exporting iron into the blood [20]. Conversely, serum iron stimulates HPC synthesis. Thus, a decrease in iron observed after the run should lead to the downregulation of HPC. Thus, it is possible that changes in HPC, EPO, and ERFE are the result of many sometimes-contradictory factors that can modify their synthesis, and vitamin D_3_ has a role in this.

There is another possible link between vitamin D_3_ and iron, which involves stress-activated protein kinases, such as c-Jun NH2-terminal kinase (JNK). Vitamin D_3_ supplementation has been demonstrated to inhibit macrophage JNK activation and decrease vascular inflammation, hypertension, and the progression of atherosclerosis in animal experimental models [21,22]. Conversely, cell culture experiments demonstrated that FER proteasomal degradation and iron-dependent ROS formation are JNK-dependent [23,24]. Additionally, an increase in heart JNK activity has been observed in animals after exercise [25]. Thus, here, we hypothesised that in athletes supplemented with vitamin D_3_, there would be a lower activation of heart JNK and iron-dependent oxidative stress, which can be manifested by lower heart damage induced by an ultramarathon. In fact, vitamin D_3_ has significant effects on cTnT, which is a marker of heart damage. There is a study showing that serum cTnT increases after a marathon [8]. Here, we observed that its serum concentration increased from 1.63 ± 0.83 to 10.42 ± 4.13 pg/mL in the placebo group and 1.70 ± 0.65 to 6.65 ± 2.90 pg/mL in the vitamin D_3_-supplemented group. Studies on haemodialysis patients demonstrated that lower 25(OH)D_3_ levels were associated with cTnT elevation [26]. Certainly, in this experimental model, we can only speculate that the observed protective effects of vitamin D_3_ on the heart are related to iron metabolism. It is also important to note that the increase in EPO concentration after the run was higher in the vitamin D_3_-supplemented group. Therefore, it cannot be excluded that the protective effect of vitamin D_3_ correlates with EPO level, which has been demonstrated to have receptors in the heart and has improved heart function in several clinical conditions [12]. To our knowledge, no study has investigated the effects of vitamin D_3_ supplementation on exercise-induced changes in cTnT.

## 5. Conclusions

In summary, this investigation uniquely examines the impact of vitamin D on exercise-induced changes in iron metabolism and heart damage. Athletes supplemented with vitamin D demonstrated higher levels of EPO and lower levels of heart damage after an ultramarathon. Changes in hormones controlling iron metabolism induced by the ultramarathon were also influenced by body iron stores. To understand why vitamin D modifies this relationship, further study is needed.

## Figures and Tables

**Table 1:** Participants’ characteristics (*n* = 35).

Variable	Supplemented Group (*n* = 16)	Placebo (Control) Group (*n* = 19)	*p*	Effect Size (η^2^)
	Mean ± SD	Mean ± SD		
Age (years)	42.40 ± 7.59	39.48 ± 6.89	0.21	0.04
Body height (cm)	175.20 ± 4.34 *	179.67 ± 4.64	0.01	0.17
Body mass (kg)	72.51 ± 6.71	76.19 ± 5.25	0.07	0.08
Body mass index (kg/m^2^)	23.24 ± 2.78	24.45 ± 1.19	0.11	0.06
Fat mass (%)	12.13 ± 3.89	12.85 ± 4.42	0.36	0.03
Baseline serum ferritin (ng/mL)	144.20 ± 43.29	149.50 ± 68.95	0.44	0.02
Baseline serum iron (µmol/L)	24.53 ± 13.16	28.27 ± 19.01	0.50	0.01

Note: *, significant difference vs. control group at *p* < 0.05.

**Table 2:** Summary of training loads during one typical week of the two periods of training (*n* = 35) [16].

		Number of Training Units per Week	CR 1 (km)	CR 2 (km)	CROSS 1 (km)	CROSS 2 (km)	Speed (km)
General preparation period	Mean	5.00	60.94	11.58	7.58	3.91	0.88
	SD	0.83	16.29	4.21	2.78	2.88	0.60
Pre-start period	Mean	5.70	67.39	13.52	14.38	5.7	1.57
	SD	0.85	11.96	2.60	4.35	2.65	0.59

Note: CR 1: 70–80% HR max—continuous running in the first intensity range (70–80% HR max); CR 2: 80–90% HR max—continuous running in the second intensity range (80–90% HR max); CROSS 1: up–downhill running at different tempos (75–85% HR max); CROSS 2: up–downhill running at different tempos (85–95% HR max); speed: 100–200 m distance running with high intensity.

**Table 3:** Characteristics of the maximum oxygen uptake capacity (VO_2_ max) in ultramarathon runners based on the Cooper test.

Variable	Supplemented Group (*n* = 16)	Placebo (Control) Group (*n* = 19)	*p*	Effect Size (η^2^)
	Mean ± SD	Mean ± SD		
VO_2_ max (mL/kg/min)	53.73 ± 6.04	54.40 ± 5.68	0.74	<0.01
Distance (km)	2.908 ± 0.263	2.939 ± 0.254	0.75	<0.01

Note: VO_2_ max—maximal oxygen uptake.

**Table 4:** Two-way ANOVA (2 groups × 3 repeated measures) of changes in erythroferrone, erythropoietin, hepcidin, and neopterin levels induced by ultramarathon.

Variable	Effect	F	df	*p*	Effect Size (η^2^)	Post hoc Outcome
Erythroferrone	GR	0.04	1, 33	0.83	<0.01	I < II < III
	UM	33.6	2, 66	0.01 *	0.49	
	GR × UM	0.55	2, 66	0.57	0.01	
Erythropoietin	GR	3.39	1, 33	0.07	0.09	
	UM	743.92	2, 66	0.01 *	0.95	I < II < III
	GR × UM	4.04	2, 66	0.01 *	0.16	C-III < S-III
Hepcidin	GR	0.94	1, 33	0.33	0.02	
	UM	12.51	2, 66	0.01 *	0.27	I < II, III
	GR × UM	0.74	2, 66	0.47	0.02	
Neopterin	GR	0.79	1, 33	0.37	0.02	
	UM	996.39	2, 66	0.01 *	0.97	I < II < III
	GR × UM	2.07	2, 66	0.13	0.05	

Study design: GR, group; C, runners who received the placebo (control group); S, runners who received a single high dose of vitamin D_3_; UM, ultramarathon; I, 24 h before the run; II, immediately after the run; and III, 24 h after the run. Significant difference detected at * *p* ≤ 0.01.

**Table 5:** Pearson’s correlation coefficient (r) of changes in ultramarathon-induced erythroferrone, erythropoietin, hepcidin, and neopterin levels with different baseline ferritin and iron serum concentrations.

Variable	Change	Supplemented Group		Placebo (Control) Group	
		Iron	Ferritin	Iron	Ferritin
Erythroferrone	Δ II–I	0.41	0.27	0.07	0.48 *
	Δ III–I	0.34	0.28	0.23	0.45 *
Erythropoietin	Δ II–I	0.13	−0.07	0.09	0.28
	Δ III–I	0.24	0.01	−0.08	0.12
Hepcidin	Δ II–I	−0.31	−0.51 *	−0.14	0.46 *
	Δ III–I	−0.04	−0.30	−0.08	0.30
Neopterin	Δ II–I	−0.09	0.31	0.13	0.42
	Δ III–I	−0.06	0.36	0.52 *	0.31

Note: Δ I–II, the difference between the values before and immediately after the ultramarathon; Δ I–III, the difference between the values before and 24 h after the ultramarathon. Significant difference at * *p* ≤ 0.05.

**Table 6:** Two-way ANOVA (2 groups × 3 repeated measures) of changes in ferritin, iron, and total and unsaturated iron-binding capacity levels induced by the ultramarathon run.

Variable	Effect	F	df	*p*	Effect Size (η^2^)	Post hoc Outcome
Ferritin	GR	1.75	1, 33	0.19	0.05	
	UM	1.06	2, 66	0.34	0.03	
	GR × UM	1.35	2, 66	0.26	0.04	
Iron	GR	0.65	1, 33	0.45	0.01	
	UM	13.25	2, 66	0.01 *	0.25	II < I, III
	GR × UM	9.11	2, 66	0.01 *	0.19	CII < CI, CIII
TIBC	GR	28.16	1, 33	0.01 *	0.42	C < S
	UM	109.51	2, 66	0.01 *	0.74	I > II < III
	GR × UM	43.62	2, 66	0.01 *	0.53	CI > CII < CIII; CI, CII < SI, SII
UIBC	GR	29.97	1, 33	0.01 *	0.44	C < S
	UM	6.64	2, 66	0.01 *	0.14	I < II, III
	GR × UM	2.07	2, 66	0.01 *	0.16	CI < CII, CII; CI < SI

Study design: TIBC, total iron-binding capacity; UIBC, unsaturated iron-binding capacity; GR, group; C, runners without vitamin D_3_ supplementation (control group); S, runners supplemented with vitamin D_3_; UM, ultramarathon; I, 24 h before the run; II, immediately after the run; and III, 24 h after the run. Significant difference detected at * *p* ≤ 0.01.

**Table 7:** Cardiac troponin T level changes induced by ultramarathon in runners supplemented with vitamin D_3_ (Mean ± SD).

Variable	Unit	Supplemented Group		Placebo (Control) Group	
		Baseline	24 h after the Run	Baseline	24 h after the Run
Cardiac Troponin T	pg/mL	1.70 ± 0.65	6.65 ± 2.90 ^#^*	1.63 ± 0.83	10.42 ± 4.13 ^#^

^#^ significant difference from baseline value at *p* ≤ 0.01; * significant difference between runners supplemented with vitamin D_3_ and runners without vitamin D_3_ supplementation at particular timepoints at *p* ≤ 0.01.

## Data Availability

The findings of this study are supported by data that can be obtained upon request due to privacy policy from the corresponding authors, J.M. and J.A.
